# Supplementary figures and images for: Time-resolved phosphoproteomics reveals scaffolding and catalysis-responsive patterns of SHP2-dependent signaling
Source: eLife. 2021 Mar 23;10:e64251. doi: 10.7554/eLife.64251 (PMC8024022; doi:10.7554/eLife.64251)

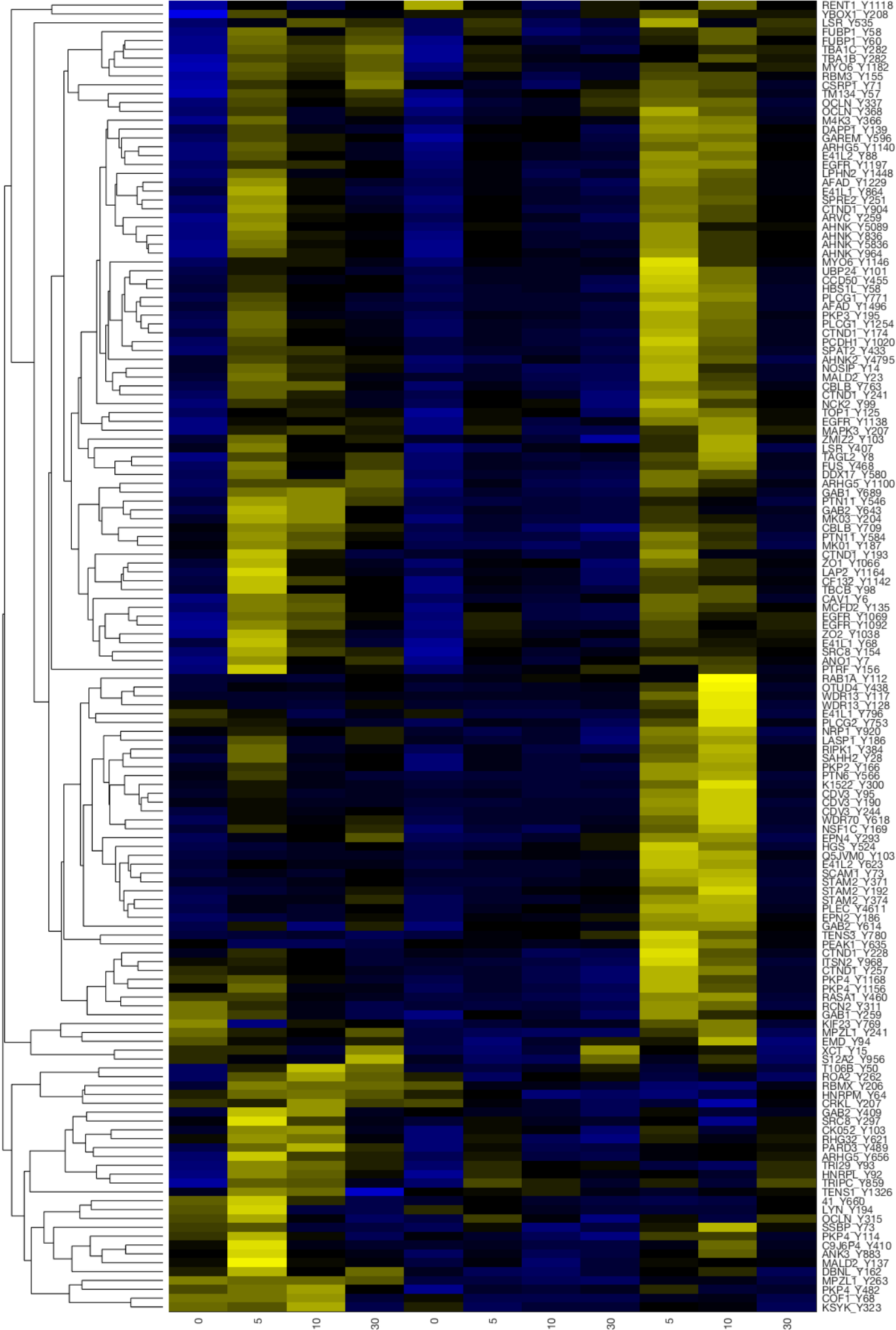

Supplement: Figure 2—figure supplement 1—source data 1. [file elife-64251-fig2-figsupp1-data1.pdf]

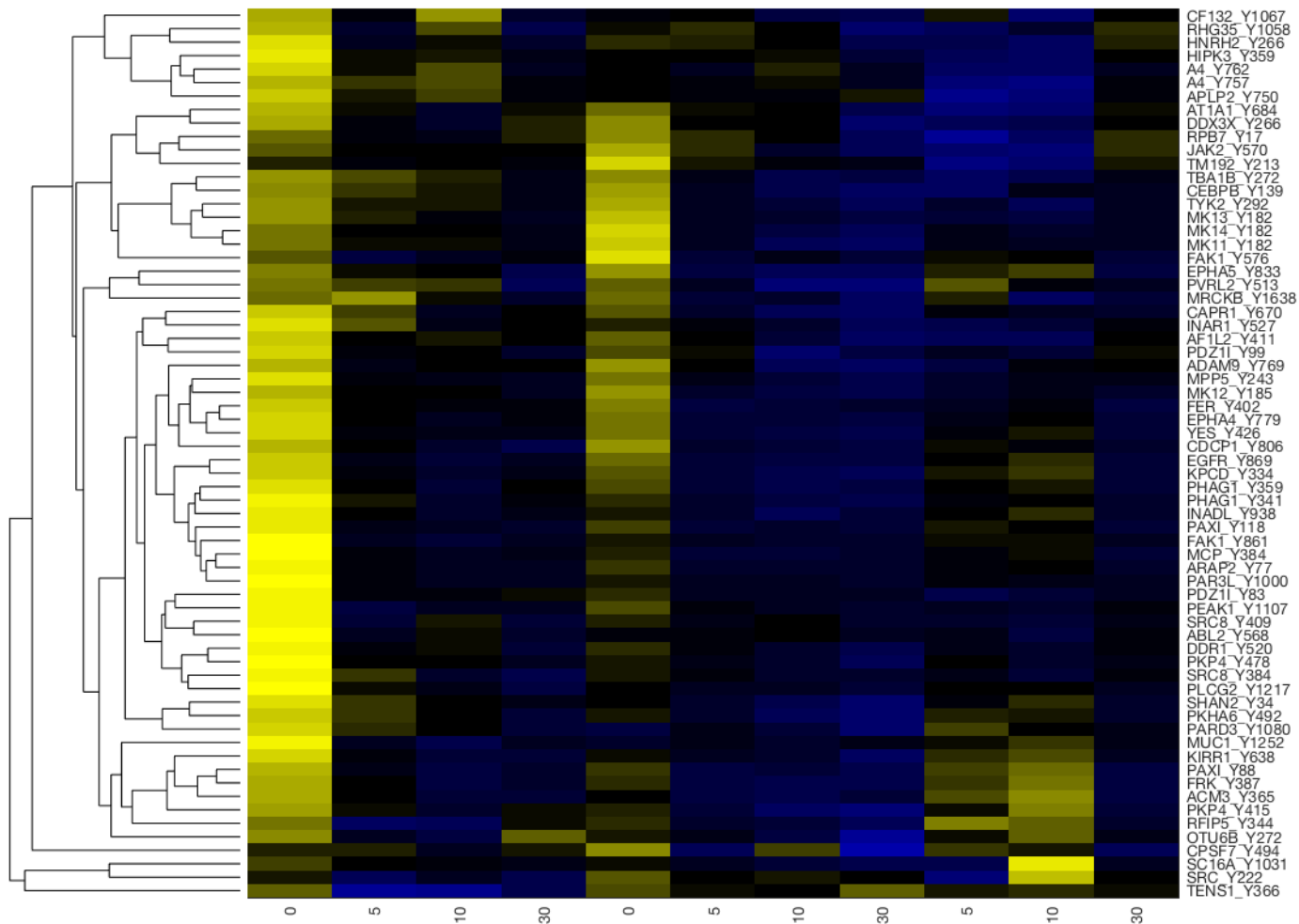

Supplement: Figure 2—figure supplement 1—source data 2. [file elife-64251-fig2-figsupp1-data2.pdf]

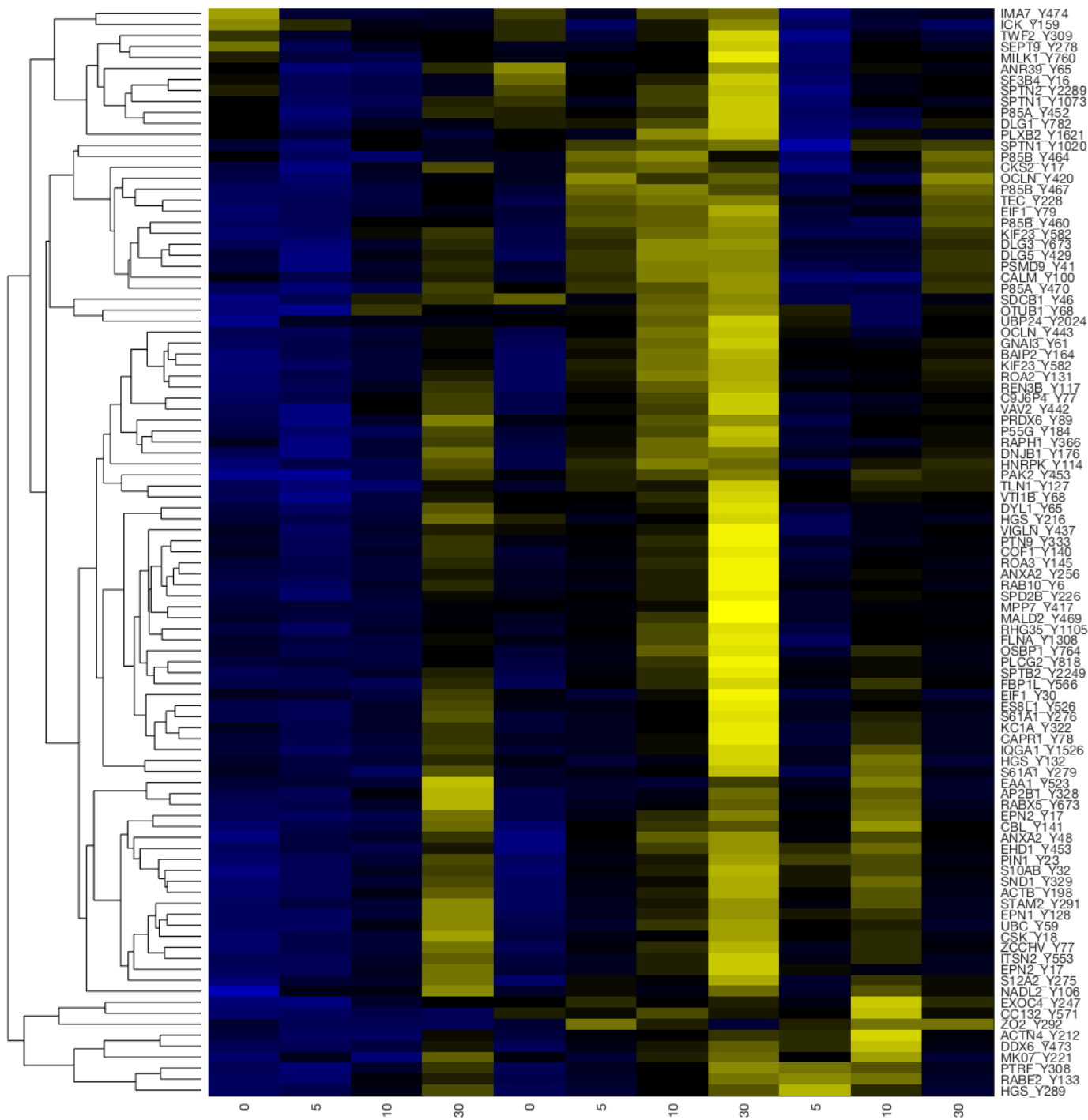

Supplement: Figure 2—figure supplement 1—source data 3. [file elife-64251-fig2-figsupp1-data3.pdf]

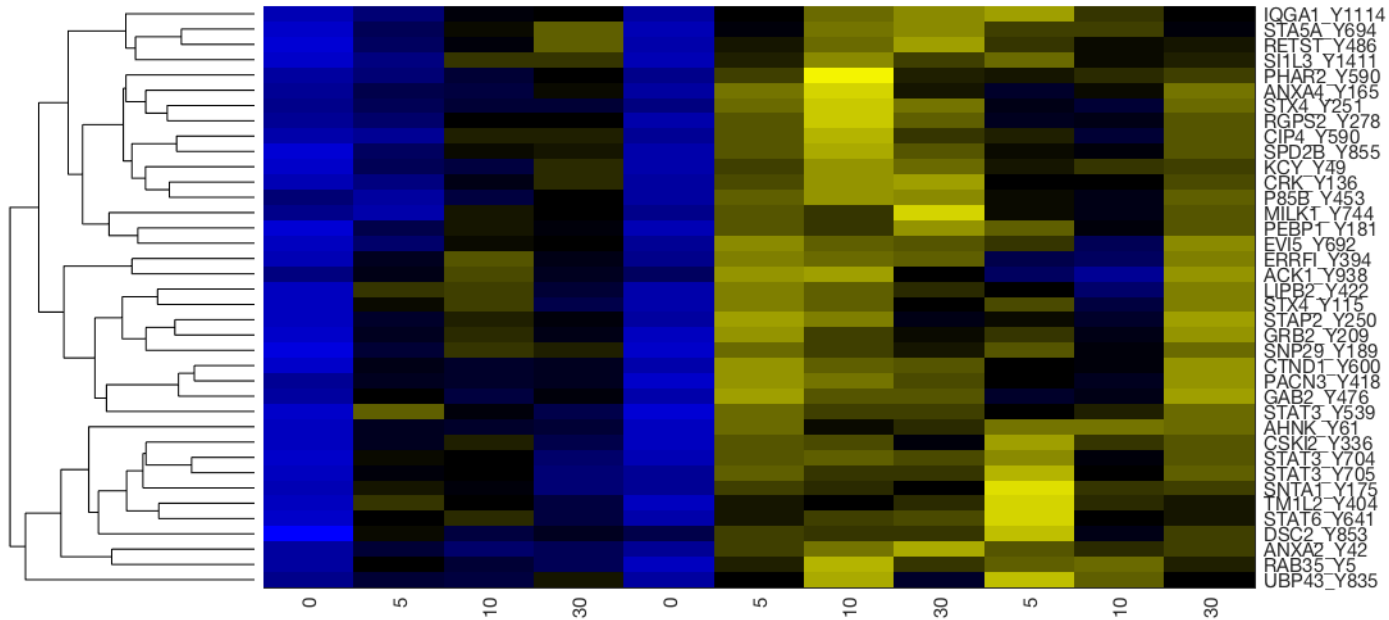

Supplement: Figure 2—figure supplement 1—source data 4. [file elife-64251-fig2-figsupp1-data4.pdf]

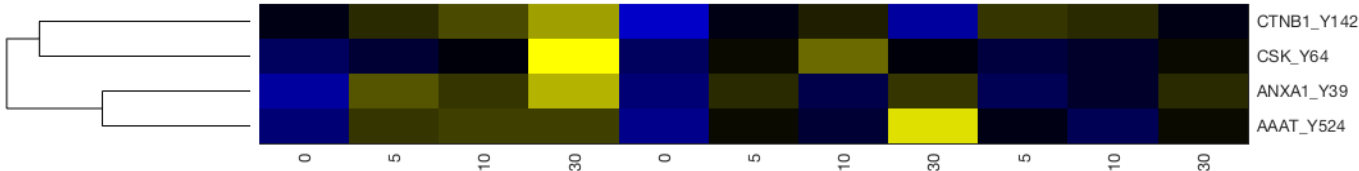

Supplement: Figure 2—figure supplement 1—source data 5. [file elife-64251-fig2-figsupp1-data5.pdf]

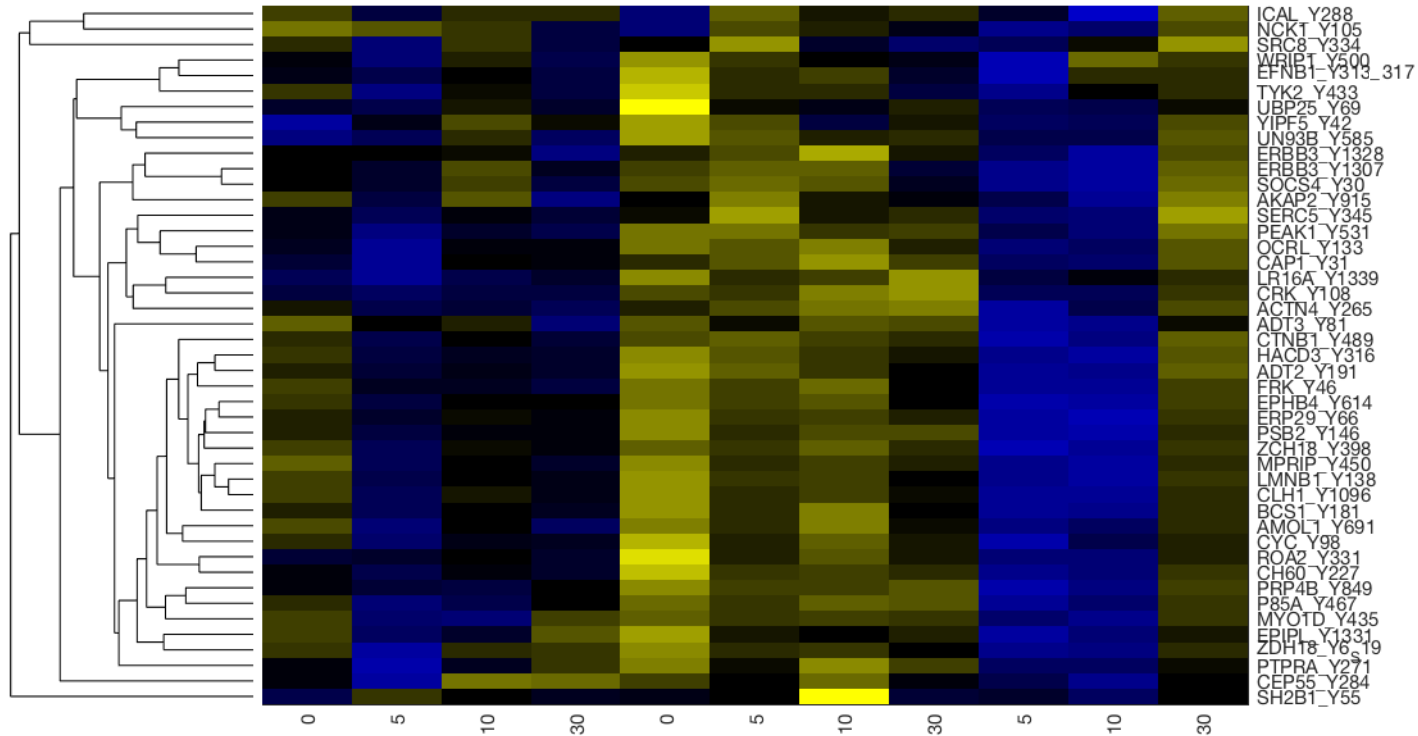

Supplement: Figure 2—figure supplement 1—source data 6. [file elife-64251-fig2-figsupp1-data6.pdf]
